# Is There a Role for Perioperative Nutritional Support in Liver Resection?

**DOI:** 10.1155/1997/64648

**Published:** 1997

**Authors:** Albert Bothe, Glenn Steele

**Affiliations:** Department of Surgery Deaconess Hospital Boston MA 02215 USA

## Abstract

*Background*: Resection of hepatocellular carcinoma is associated with high rates of morbidity and mortality. Since intensive nutritional support can reduce the catabolic response and improve protein synthesis and liver regeneration, we performed a prospective study to investigate whether perioperative nutritional support could improve outcome in patients undergoing hepatectomy for hepatocellular carcinoma.

*Methods*: We studied 124 patients undergoing resection of hepatocellular carcinoma. Sixty-four patients (39 with cirrhosis, 18 with chronic active hepatitis, and 7 with no associated liver disease) were randomly assigned to receive perioperative intravenous nutritional support in addition to their oral diet, and 60 patients (33 with cirrhosis, 12 with chronic active hepatitis, and 15 with no associated liver disease) were randomly assigned to a control group. The perioperative nutritional therapy consisted of a solution enriched with 35 percent branched chain amino acids, dextrose, and lipid emulsion (50 percent medium-chain trigylcerides) given intravenously for 14 days perioperatively).

*Results*: There was a reduction in the overall postoperative morbidity rate in the perioperative-nutrition group as compared with the control group (34 percent vs. 55 percent; relative risk, 0.66; 95 percent confidence interval, 0.45 to 0.96), predominantly because of fewer septic complications (17 percent vs. 37 percent; relative risk, 0.57; 95 percent confidence interval, 0.34 to 0.96). There were also a reduction in the requirement for diuretic agents to control ascites (25 percent vs. 50 percent; relative risk, 0.57; 95 percent confidence interval, 0.37 to 0.87), less weight loss after hepatectomy (median loss, 0 kg vs. 1.4 kg; *P*=0.01), and less deterioration of liver function as measured by the change in the rate of clearance of indocyanine green (–2.8 percent vs. –4.8 percent at 20 minutes, *P*=0.05). These benefits were seen predominantly in the patients with underlying cirrhosis who underwent major hepatechtomy. There were five deaths during hospitalization in the perioperativenutrition group, and nine in the control group (P not significant).

*Conclusions*: Perioperative nutritional support can reduce complications after major hepatectomy for hepatocellular carcinoma associated with cirrhosis.


*HPB Surgery*, 1997, Vol.10, pp. 177–193
Reprints available directly from the publisher
Photocopying permitted by license only

© 1997 OPA (Overseas Publishers Association)
Amsterdam B.V. Published in The Netherlands
by Harwood Academic Publishers
Printed in India

### HPB INTERNATIONAL

**EDITORIAL & ABSTRACTING SERVICE**

JOHN TERBLANCHE, EDITOR
Department of Surgery,
Medical School · Observatory 7925
Cape Town · South Africa
Telephone: 27-21-406-6204
Telefax 27-21-448-6461
E-mail: JTER BLAN@UCT GSHI.UCT.AC.2A

### IS THERE A ROLE FOR PERIOPERATIVE NUTRITIONAL SUPPORT IN LIVER RESECTION?

### ABSTRACT

*Fan, S-T., Lo, C-M., Lao, E. C. S., Chu, K-M., Liu, C-L. and Wong, J. (1994) Perioperative nutritional support in patients undergoing hepatectomy for hepatocellular carcinoma. New England Journal of Medicine; 331: 1547–1552.*

***Background:*** **Resection of hepatocellular carcinoma is associated with high rates of morbidity and mortality. Since intensive nutritional support can reduce the catabolic response and improve protein synthesis and liver regeneration, we performed a prospective study to investigate whether perioperative nutritional support could improve outcome in patients undergoing hepatectomy for hepatocellular carcinoma.**
***Methods:*** **We studied 124 patients undergoing resection of hepatocellular carcinoma. Sixty-four patients (39 with cirrhosis, 18 with chronic active hepatitis, and 7 with no associated liver disease) were randomly assigned to receive perioperative intravenous nutritional support in addition to their oral diet, and 60 patients (33 with cirrhosis, 12 with chronic active hepatitis, and 15 with no associated liver disease) were randomly assigned to a control group. The perioperative nutritional therapy consisted of a solution enriched with 35 percent branched chain amino acids, dextrose, and lipid emulsion (50 percent medium-chain trigylcerides) given intravenously for 14 days perioperatively).**
***Results:*** **There was a reduction in the overall postoperative morbidity rate in the perioperative-nutrition group as compared with the control group (34 percent vs. 55 percent; relative risk, 0.66; 95 percent confidence interval, 0.45 to 0.96), predominantly because of fewer septic complications (17 percent vs. 37 percent; relative risk, 0.57; 95 percent confidence interval, 0.34 to 0.96). There were also a reduction in the requirement for diuretic agents to control ascites (25 percent vs. 50 percent; relative risk, 0.57; 95 percent confidence interval, 0.37 to 0.87), less weight loss after hepatectomy (median loss, 0 kg vs. 1.4 kg; *P*=0.01), and less deterioration of liver function as measured by the change in the rate of clearance of indocyanine green (−2.8 percent vs. −4.8 percent at 20 minutes, *P*=0.05). These benefits were seen**

**predominantly in the patients with underlying cirrhosis who underwent major hepatechtomy. There were five deaths during hospitalization in the perioperative-nutrition group, and nine in the control group (P not significant).**
***Conclusions:*** **Perioperative nutritional support can reduce complications after major hepatectomy for hepatocellular carcinoma associated with cirrhosis. (N Engl J Med 1994; 331: 1547–52.)**

KEY WORDS: Liver resection, hyperalimentation, cirrhosis

### PAPER DISCUSSION

Since the study by Muller *et al.*[1] more than a decade ago showing the benefit of parenteral feeding in the perioperative period, efforts have continued to refine the indications for nutritional support in the surgical patient. Fan and his colleagues[2] have identified another such specific group of patients. However, several recent reports prompt caution before extrapolating the finding to other patients. The large, prospective VA Co-operative Study[3] showed, by subset analysis, that only severely malnourished surgical patients benefit from parenteral nutrition support. In another study, Sandström[4] was not able to demonstrate improved outcome in a large group of 300 patients undergoing major general surgical procedures, including 33 hepatectomies. Brennan *et al.*[5] showed no benefit of adjuvant parenteral nutrition in patients undergoing major pancreatic resections. Further, that study showed more complications in the TPN group.

Fan has studied the impact of TPN on postoperative complications after hepatectomy which has a historical high morbidity rate in his patient population. However, more recent publications by the same group[6,7] report improved morbidity and mortality rates, emphasizing the importance of concurrent controls.

Like the VA Cooperative Study, patients in Fan's study had access to regular oral diets in the pre-operative period. Thus, the parenteral nutrition group is compared to a control group with some level of undocumented nutrient intake prior to surgery. The entire preoperative IV nutrient feeding appears to have been provided to the treatment group in an overnight bolus, in addition to daytime oral feedings. Nutritional assessment parameters are reported at randomization, but not prior to surgery which is after seven days of feeding.

Analysis of the postoperative morbidity shows that virtually all of the improvement in incidence of septic complications in the treatment group is for pulmonary infections, a difference not found in other recent studies. It is also of interest that 4 of the 5 subphrenic abscesses in the control group were lethal compared with no deaths in the 4 patients in the treatment group with the same complication. The development of hepatic coma postoperatively appears to have been uniformly lethal, regardless of nutritional support, though the definition of coma is not provided. The high rate of need for diuretic agents in the control group posed a clinical problem, since the patients were simultaneously receiving 1.75 liters of crystalloid per day to match the fluid volume of the TPN group. The statistically higher glucose levels in the treatment group on days 2–5 may have afforded an osmotic diuretic effect, especially noting the wide upper ranges of hyperglycemia reported.

Subgroup analysis shows a statistical reduction in postoperative morbidity for the treatment group if cirrhosis was present or if the patient underwent major hepatectomy. The small size of the subgroups obscures the statistical benefit to cirrhotic patients if the procedure is limited to a minor hepatectomy.

The fact that neither mortality nor length-of-stay were improved by nutritional support implies that the septic complications which were more frequent in the control group had limited clinical significance. Rather than advocating that perioperative intravenous nutritional support be used for all patients with hepatocellular carcinoma undergoing hepatectomy, such nutritional intervention might be limited to cirrhotic patients who are to have major resections or who have had significant preoperative weight loss.

Further clinical studies might address the intriguing animal model results of Delaney *et al.*[8] suggesting that enteral feeding is of greater benefit after hepatectomy than parenteral feeding. Meanwhile, the work by Fan and his colleagues is another bit of important information in defining the disease-specific, procedure-specific and nutritional-status-specific indications to be used in initiating an effective feeding strategy.

### REFERENCES

1. Muller, J. M., Dienst, C., Brenner, U. and Pichlmaier, H. (1982). Preoperative parenteral feeding in patients with gastrointestinal carcinoma, *Lancet*, **1**, 68–71.

2. Fan, S. T., Lo, C. M., Lai, E., Chu, K. M., Liu, C. L. and Wong, J. (1994). Perioperative nutritional support in patients undergoing hepatechtomy for hepatocellular carcinoma, *NEJM*, **331**, 1547–1552.
3. Veterans' Affairs Total Parenteral Nutrition Cooperative Study Group (1991). Perioperative total parenteral nutrition in surgical patients, *NEJM*, **325**, 525–532.
4. Sandström, R., Drott, C. and Hyltander, A., *et al.* (1993). the effect of postoperative intravenous feeding (TPN) on outcome following major surgery evaluated in a randomized study, *Annals of Surgery*, **217**, 185–195.
5. Brenan, M. F., Pisters, P. W. T. and Posner, M., *et al.* (1994). A prospective randomized study of total parenteral nutrition after major pancreatic resection for malignancy, *Annals of Surgery*, **222**, 436–444.
6. Fan, S.T., Lai, E. C. and Lo, C. M., *et al.* (1995). Hospital mortality of major hepatectomy for hepatocellular carcinoma associated with cirrhosis, *Archives of Surgery*, **130**, 198–203.
7. Lai, E. C., Fan, S. T. and Lo, C. M., *et al.* (1995). Hepatic resection for hepatocellular carcinoma, an audit of 343 patients, *Annals of Surgery*, **221**, 291–298.
8. Delany, H. M., John, J. and Teh, E. L., *et al.* (1994). Contrasting effects of identical nutrients given parenterally or enterally after 70% hepatectomy, *American Journal of Surgery*, **167**, 135–143.

Albert Bothe Jr. and Glenn Steele Jr.
Department of Surgery
Deaconess Hospital
Boston, MA 02215
United States of America

### STENT OR SURGERY FOR MALIGNANT LOW BILEDUCT OBSTRUCTION?

### ABSTRACT

*Smith, A.C., Dowsett, J.F., Russell, R.C.G., Hatfield, A.R.W. and Cotton, P.B. (1994) Randomised trial of endoscopic stenting versus surgical bypass in malignant low bileduct obstruction. The Lancet; 344: 1655–1660.*

**The development of non-surgical techniques for the relief of malignant low bileduct obstruction has cast doubt on the best way of relieving jaundice, particularly in patients fit for surgery whose life expectancy is more than a few weeks.**

**We did a randomised prospective controlled trial comparing endoscopic stent insertion and surgical biliary bypass in patients with malignant low bileduct obstruction. 204 patients were randomised (surgery 103, stent 101); 3 subsequently proved to have benign disease and were excluded, leaving 101 surgical and 100 stented patients for assessment. Technical success was achieved in 94 surgical and 95 stent patients, with functional biliary decompression obtained in 92 patients in both groups. In stented patients, there was a lower procedure-related mortality (3% *vs* 14%, $p$=0.01), major complication rate (11% *vs* 29%, $p$=0.02), and median total hospital stay (20 *vs* 26 days, $p$=0.001). Recurrent jaundice occurred in 36 stented patients and 2 surgical patients. Late gastric outlet obstruction occurred in 17% of stented patients and 7% of the surgical group. Despite the early benefits of stenting there was no significant difference in overall survival between the two groups (median survival: surgical 26 weeks; stented 21 weeks; $p$=0.065).**

**Endoscopic stenting and surgery are effective palliative treatments with the former having fewer early treatment-related complications and the latter fewer late complications.**

**Lancet 1994; 344: 1655–60**

KEYWORDS: Endoprothesis, Biliary enteric bypass, bileduct, carcinoma pancreas

